# Resistance models to EGFR inhibition and chemotherapy in non-small cell lung cancer via analysis of tumour size dynamics

**DOI:** 10.1007/s00280-019-03840-3

**Published:** 2019-04-24

**Authors:** Hitesh B. Mistry, Gabriel Helmlinger, Nidal Al-Huniti, Karthick Vishwanathan, James Yates

**Affiliations:** 1 Division of Pharmacy, University of Manchester, Manchester, UK; 2 Quantitative Clinical Pharmacology, Early Clinical Development, IMED Biotech Unit, AstraZeneca, Cambridge, USA; 3 DMPK, IMED Oncology, AstraZeneca, Cambridge, UK

**Keywords:** Heterogeneity, Imaging, Non-small cell lung cancer, Pharmacology

## Abstract

**Purpose:**

Imaging time-series data routinely collected in clinical trials are predominantly explored for covariates as covariates for survival analysis to support decision-making in oncology drug development. The key objective of this study was to assess if insights regarding two relapse resistance modes, de-novo (treatment selects out a pre-existing resistant clone) or acquired (resistant clone develops during treatment), could be inferred from such data.

**Methods:**

Individual lesion size time-series data were collected from ten Phase III study arms where patients were treated with either first-generation EGFR inhibitors (erlotinib or gefitinib) or chemotherapy (paclitaxel/carboplatin combination or docetaxel). The data for each arm of each study were analysed via a competing models framework to determine which of the two mathematical models of resistance, de-novo or acquired, best-described the data.

**Results:**

Within the first-line setting (treatment naive patients), we found that the de-novo model best-described the gefitinib data, whereas, for paclitaxel/carboplatin, the acquired model was preferred. In patients pre-treated with paclitaxel/carboplatin, the acquired model was again preferred for docetaxel (chemotherapy), but for patients receiving gefitinib or erlotinib, both the acquired and de-novo models described the tumour size dynamics equally well. Furthermore, in all studies where a single model was preferred, we found a degree of correlation in the dynamics of lesions within a patient, suggesting that there is a degree of homogeneity in pharmacological response.

**Conclusions:**

This analysis highlights that tumour size dynamics differ between different treatments and across lines of treatment. The analysis further suggests that these differences could be a manifestation of differing resistance mechanisms.

**Electronic supplementary material:**

The online version of this article (10.1007/s00280-019-03840-3) contains supplementary material, which is available to authorized users.

## Introduction

Assessment of a new treatment in oncology involves recording changes in tumour burden, measured via imaging, and is expressed as tumour size metrics, within a patient and over time. These data are then reduced to assess clinical response based upon the Response Evaluation Criteria In Solid Tumours (RECIST) [1, 2]. The criterion categorises multiple tumour lesions within a patient into either target or non-target lesions, based on how easy they are to measure. Only drug effect on the target lesions is recorded quantitatively over time, through the sum-of-longest-diameters (SLD) metric, while effects on non-target lesions are recorded qualitatively. The information on drug effect on target and non-target lesions together with whether a new lesion occurs is used to place patients into one of four response categories: complete response (CR), partial response (PR), stable disease (SD), or progressive disease (PD). It is this PD category which is of interest when considering resistance, as is how the depth of response, CR/PR/SD, relates to time to PD. A patient may radiologically progress via an increase in SLD, or a non-target lesion growth, or due to appearance of a new lesion. Therefore, there are several progression groups which may potentially exist, if we consider all progression combination possibilities. Any one of these possibilities can be considered a sign of resistance to treatment. However, only one of the imaging variables, SLD, provides us with quantitative time-series data and, for this reason, we focused our attention on this particular variable. Models of tumour size dynamics exist in the literature [3] with some reflecting reduced effectiveness over time; however, no study to our knowledge examines the potential sources of resistance.

**Fig. 1:**
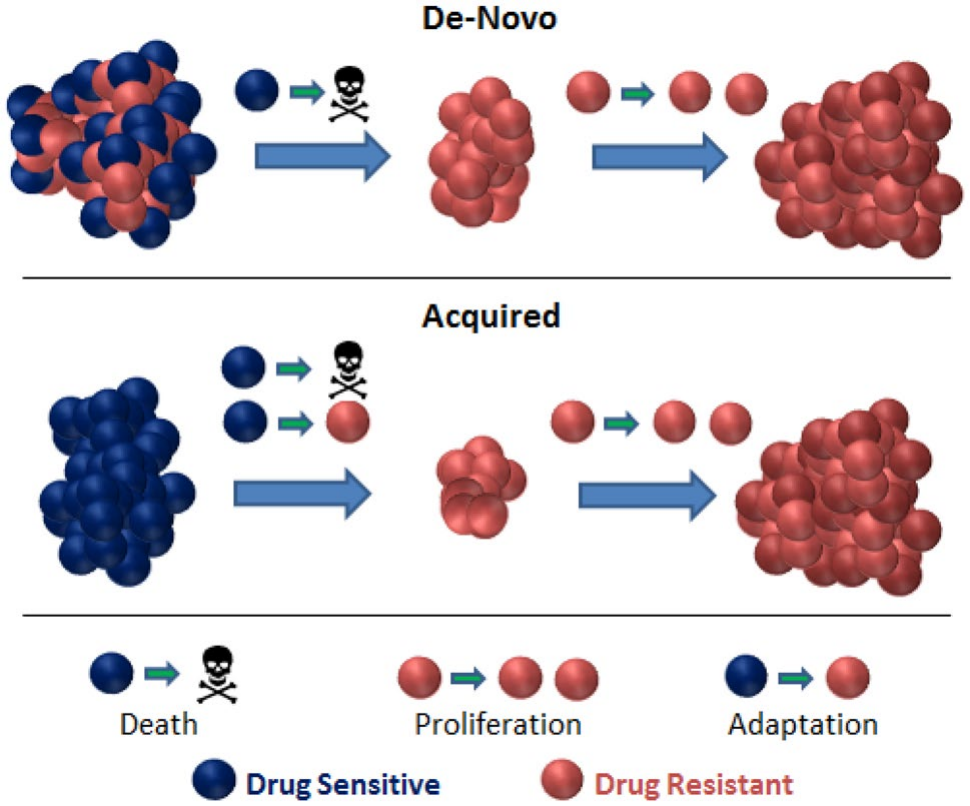
Pictorial representation of the mathematical models of resistance considered here: de-novo and acquired. In the de-novo model, drug treatment is assumed to select out a specific resistant clone. Once treatment is applied, the drug sensitive population dies at a rate, d, whereas the drug resistant cells continue to proliferate at a rate, g. In the acquired model, drug treatment leads to an adaptation of the initial tumour cell population. Once treatment is applied, a certain proportion of cells die at a rate, d, others adapt at a rate, c, to become drug resistant and subsequently proliferate at a rate, g

The purpose of this research was to explore the dynamics of relapsing individual lesions using an approach that combines mathematical biology, applied statistics, and clinical data. The mathematical biology component was used to derive two common models of resistance as described by Hata et al. [4]: (i) de-novo—pre-existing resistant clone is selected out during treatment; (ii) acquired—proportion of tumour cells adapt, during treatment, to become resistant [3] after an initial response (Fig. 1). In the case of EGFR inhibitors, a common mechanism of resistance in NSCLC is the acquisition of the T790 M “gatekeeper” mutation on the egfr gene; however, it is not clear whether this exists prior to treatment. The models developed are simple enough, such that they can be easily parameterised using routinely collected data. The resultant models were placed within a statistical population analysis framework to determine how well they describe the data. Therefore, we approached the data with predefined hypotheses based on our biological understanding rather than allowing the data to guide our choice of a structural model. In addition to the modelling component described above, we also considered visualising the dynamics of tumour size changes to highlight differences in dynamics for different treatments, without the aid of fitting a model to data.

The approach described was applied to ten arms of nine clinical studies involving the first-generation EGFR inhibitors (gefitinib and erlotinib) as well as chemotherapies (paclitaxel/carboplatin combination and docetaxel), across both first- (treatment naive) and second-line settings in non-small cell lung cancer (NSCLC). The modelling approach which we undertook allowed us to answer the following questions: (1) are the resistance hypotheses different for EGFR inhibitors versus chemotherapy? (2) does the type of resistance dictate the growth rate of the emerging resistant clone? (3) how large is the resistant fraction under the de-novo hypothesis and how does it vary across tumours versus across patients? The answers to these questions will add to our knowledge about what information can be gained from routine imaging data regarding resistance patterns.

## Methods

### Patients and data

Target lesion measurements of patients from 10 arms of 9 clinical studies were collected. References of the studies used can be found in the first column of Table 1. All data except for the studies using gefitinib were available in ProjectDataSphere [5, 6]. Gefitinib data were obtained from AstraZeneca. Only patients who had a response to treatment, i.e., those who were classified as CR, PR, or SD at the first visit, were taken forward for further analysis. This allowed us to look at response followed by resistance (hence, patient relapse), the objective of this study. On average, patients had 4–5 CT scans measured every 6–8 weeks and were followed on treatments. Gefitinib was dosed orally 250 mg daily, whereas the chemotherapy was dosed every 3 weeks; for further details of the studies, we refer the reader to the original studies (note that there was no information within the data sets on whether the tumour size measurements were below the limit of quantification).

**Table 1:** Characteristics of the clinical trials used within this analysis

References	Line of therapy	Treatment	Patient *N* (lesions *N*)	PFS median (95% CI)	No. prog. events (deaths)	BSL ILD (mm) Median (IQR)
IPASS Phase III [12]	First	Gefitinib (unselected)	338 (781)	6.9 (6.7–8.1)	261 (8)	25 (17–38)
	First	Paclitaxel/carboplatin	427 (1066)	6.6 (6.1–6.9)	371 (14)	25 (18–38)
ABRAXANE Phase III [13]	First	Paclitaxel/carboplatin	414 (1664)	7 (6.4–7.3)	299 (6)	22 (15–35)
IFUM Phase IV [14]	First	Gefitinib (selected)	92 (294)	10.3 (8.6–13.8)	43 (1)	26 (18–40)
ZEST Phase III [15]	First	Erlotinib (unselected)	213 (534)	7.3 (5.5–7.5)	164 (14)	26 (16–35)
SUNITINIB Phase III [16]	Second	Erlotinib (unselected)	193 (551)	6.4 (5.6–7.4)	141 (11)	23 (16–37)
IDEAL1 Phase II [17]	Second/third	Gefitinib (unselected)	117 (240)	4.2 (3.7–5.1)	62 (0)	28 (18–44)
INTEREST Phase III [18]	Second	Docetaxel	278 (800)	4.8 (4.3–5.3)	216 (13)	23 (15–40)
ZODIAC Phase III [19]	Second	Docetaxel	337 (900)	5.4 (4.9–5.6)	293 (20)	23 (16–37)
VITAL Phase III [20]	Second	Docetaxel	282 (898)	5.7 (5.4–6.8)	247 (15)	22 (15–35)

Unselected/selected defines whether genomic criteria were used for patient selection

*PFS* progression-free survival, *95% CI* 95 percent confidence interval, *BSL* baseline, *ILD* individual longest diameter, *IQR* inter-quartile range

### Resistance models

The time-series models used to analyse individual lesion dynamics are graphically depicted in Fig. 1. The radiologically measured longest diameter is used as a surrogate for tumour size and so tumour cell population. For the *de*-*novo* resistance model, four parameters needed to be estimated: (i) initial size of the drug sensitive part of a lesion, *Y*_*1*_*(0)*; (ii) initial size of the drug resistant part, *Y*_*2*_*(0)*; (iii)–(iv) the corresponding rates of decay, *d*, and of growth, *g*. For the *acquired* resistance model, again, four parameters needed to be estimated: (i) the initial longest diameter, *Y(0)*; the adaptation (mutation) rate, *c*; (iii) the decay rate, *d*; (iv) the growth rate, *g*. Therefore, the two mathematical models of shared common parameters and structure, with one difference; however: one model allowed for the selection of a pre-existing resistant clone during treatment; the other allowed for conversion to resistant disease during treatment (without further granularity in the data, we were not able to model a combination of both scenarios). Full derivation of model equations for both de-novo and acquired resistance models can be found in the supplemental methods, analytical solutions are provided in the following:

$${\text{De-novo:}}\, Y\left( t \right) = Y_{1} \left( 0 \right){\text{e}}^{{ - {\text{dt}}}} + Y_{2} \left( 0 \right){{\text{e}}^{\text{gt}}}$$

$${\text{Acquired:}}\, Y\left( t \right) = Y_{1} \left( 0 \right)\left( {1 - \frac{c}{d + c + g}} \right){{\text{e}}^{{ - \left( {d + c} \right)t}}} + \frac{{cY_{1} \left( 0 \right)}}{d + c + g}{{\text{e}}^{\text{gt}}} .$$

The two models are essentially sum of exponentials but with different parameterisations.

The resultant models were fit to the longest diameter data by placing them within a non-linear mixed-effect framework. All parameters were assumed to follow a log-normal distribution. Residual errors were assumed to be additive. Diagnostic plots (standardised residuals versus individual fitted; individual fitted versus observed; and standardised residuals over time) and parameter values for the final models are reported in the supplemental results.

### Model assessment

A model competition-based approach, as used in survival prediction competitions [7], was implemented to assess which model best-described the time-series of individual lesion longest diameters in the following way. 1000 bootstrap samples [8] of the exact sample size of the original study arms were generated from the original data by sampling at patient level. Both models were fitted to each sample with knowledge of which lesion belonged to which patient. Bayesian Information Criterion (BIC), which provides information on the degree to which the model describes the data, was recorded for each model. Using these values across all samples, we calculated Bayes Factors to assess how likely one model was, over another, at describing the data [7] for that particular study arm. Bayes Factors are calculated in the following way which is based on the method used by Guinney et al. [7] Two models M1 and M2 are fit to a bootstrap sample Bi (with i varying from 1 to 1000). Therefore, for each sample, i, we generate a Bayesian Information Criteria value for both models, BIC-M1i and BIC-M2i, for, respectively, models M1 and M2. To generate a Bayes’ factor, we calculate the number of samples for which BIC-M1i > BIC-M2i and divide this quantity by the number of samples for which BIC-M1i < BIC-M2i. This then provides us with a score of how often model M1 provides a better fit over model M2 across the bootstrap samples. Thus, if the value of Bayes’ factor is 1, then both models are equally as good as each other in describing the data. If the value is different to 1, then one model is preferred over the other; the larger the deviation from 1, the stronger the evidence for one model over another. From the resultant resistance model, we assessed the effect of treating lesions independently from each other versus accounting for which lesion belonged to which patient, nesting between- and within-patient random effects, using the same mixed-effects approach.

### Autoregressive time-series analysis

In addition to performing a model-based analysis, we also assessed whether differences in dynamics between study arms could be visualised from the raw data. Our approach involved the use of a first-order autoregressive (AR) model [9], *Y*_*n*+*1*_ =* αY*_*n*_, where *Y*_*n*_ is the size of an individual lesion at visit *n*, and hence, *α* represents the relative change in individual lesion size between current and previous visits. For each lesion in each patient in each study arm, we generated a series of *α* values over time that described the relative change in individual lesion size from one visit to the next. To visualise how these *α* values changed over time, we employed the following approach. For the study arms which we wanted to compare, we visualised the frequency of *α* values over time, to ensure that data collection times were consistent between the study arms being compared. If frequency distributions overlapped, it implied data collection between the two study arms was independent of time. We then proceeded to calculate an ROC AUC value (area under the receiver-operating characteristic curve) at each post-baseline visit, which calculates how well the *α* values over two consecutive visits could discriminate between those visits. The series of ROC AUC values together with their 95% confidence intervals were plotted over visit number from the study arms of interest and compared visually. The resultant graph highlighted how tumour size changed from one visit to the next and how this differed between two treatments.

All analyses were performed in R v3.1.1. [10] The mixed-effects modelling analysis was conducted using the nlme package [11].

## Results

### Characteristics of patients and studies

There are two notable observations surrounding the clinical study characteristics (Table 1). First, the numbers of deaths due to progression are low; less than 10% in each study. This suggests that patients who have a CR, PR, or SD response at their first visit are unlikely to die before their disease radiologically progresses. Given that imaging time-series ceased to be collected once a patient’s disease had progressed, these data show that the time-series drop-out mechanism is not informative of survival. Rather, it is informative of when a patient stops taking one treatment and moves on to the next.

Second, the distribution of the longest diameter sizes across all lesions within a study is consistent across all studies. This shows that there is a degree of consistency, based on the initial size, in the choice of lesions by radiologists across all these studies.

### Resistance models

**Fig. 2:**
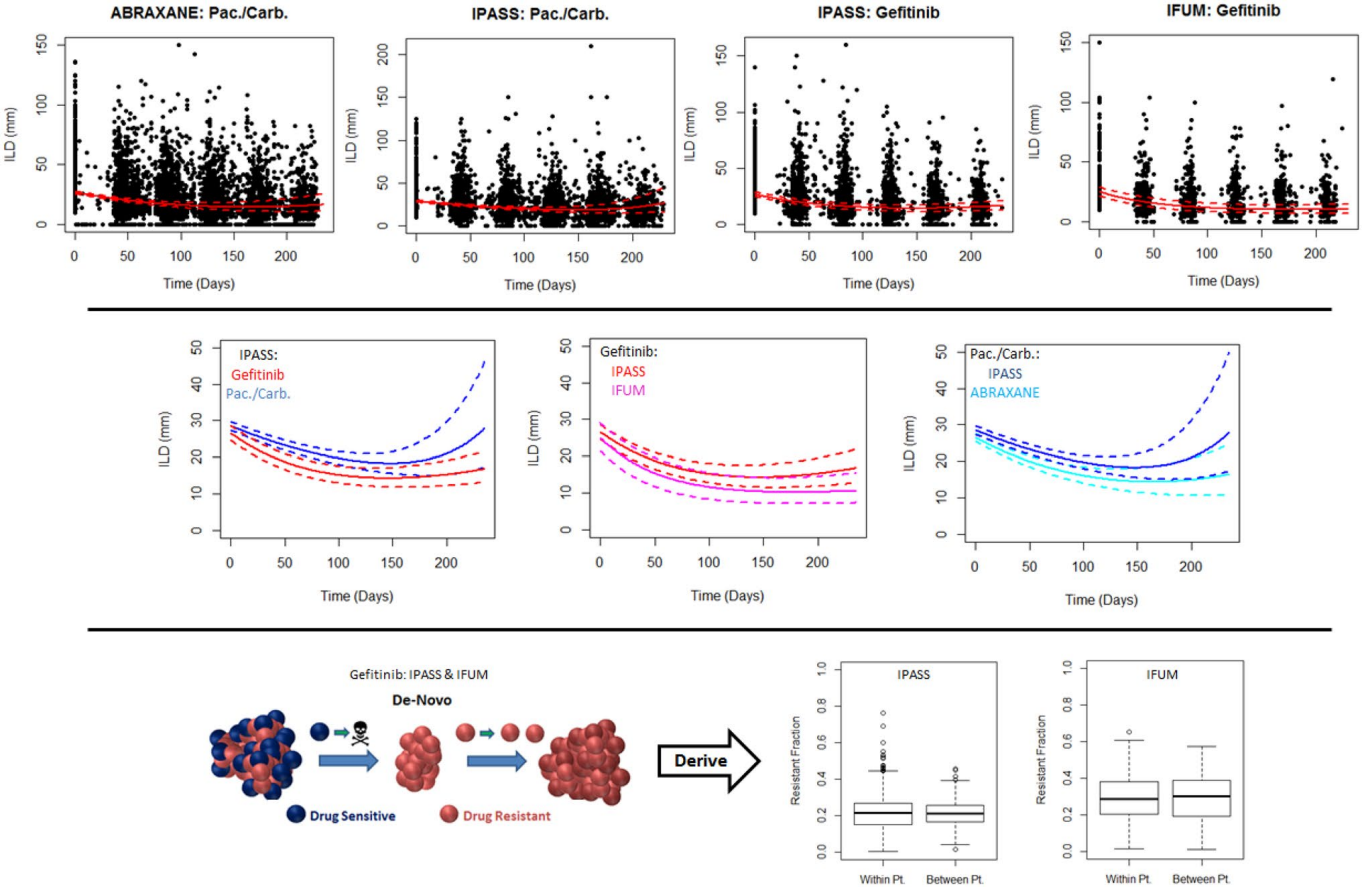
Plots showing the resistance modelling results within the treatment naive (first-line) setting. Top row: plots of raw individual longest diameter data over time (black dots) together with mean model simulations (solid lines) and 95% confidence intervals (dashed lines) for first-line treatments. Middle row: comparison of the dynamics via model simulations between treatments in the same study and the same treatments across different studies for first-line treatments. Bottom row: boxplots showing the within- (Within Pt.) and between-patient (Between Pt.) variability in the resistant fraction for gefitinib in the IFUM and IPASS studies

Results of the resistance competition between the *de*-*novo* and *acquired* models show a degree of consistency for the same drug within the same line of treatment (Table 2). In the first-line (treatment naive) setting, we found that, for paclitaxel/carboplatin, the *acquired* model was preferred, whereas, for gefitinib, the *de*-*novo* model was preferred. Simulated mean profiles, from the preferred models, with 95% CI overlaid on top of the raw data can be seen in the top row of Fig. 2. A comparison of those simulated profiles between gefitinib and paclitaxel/carboplatin can be seen in the middle row of Fig. 2. The comparison confirms the resistance competition findings: tumour size dynamics differ between gefitinib and paclitaxel/carboplatin for treatment naive patients. We see that gefitinib shrinks tumours quicker than paclitaxel/carboplatin, log*(d)* is − 4.47 (95% CI − 4.58, − 4.35) for gefitinib versus − 5.47 (95% CI − 5.56, − 5.38) for paclitaxel/carboplatin. In addition, the rate of re-growth appears to be faster under paclitaxel/carboplatin than gefitinib (log*(g)* is − 5.42 (95% CI − 5.56, − 5.28) for gefitinib versus − 4 (95% CI − 4.11, − 3.88)for paclitaxel/carboplatin), which is further confirmed by analysing the decay and growth rate parameters (Supplemental Tables S4–S7). Furthermore, these results appear to be consistent across two independent studies for both treatments, middle row of Fig. 2. Thus, the results suggest that resistance under gefitinib treatment leads to a less aggressive resistant clone than resistance under paclitaxel/carboplatin in the treatment naive setting.

**Table 2:** Resistance model results

References	Line of therapy	Treatment	Resistance	Hierarchy
			Bayes’ factor (< 1/3: acquired; > 3: de-novo)	Bayes’ factor (< 1/3: independent; > 3: correlated)
*Bootstrapping results*				
IPASS Phase III [12]	First	Gefitinib (unselected)	19.4	>30
	First	Paclitaxel/carboplatin	0.05	>30
ABRAXANE Phase III [13]	First	Paclitaxel/carboplatin	0.06	>30
IFUM Phase IV [14]	First	Gefitinib (selected)	>30	>30
ZEST Phase III [15]	Second	Erlotinib (unselected)	1.12	NA
SUNITINIB Phase III [16]	Second	Erlotinib (unselected)	0.41	NA
IDEAL1 Phase II [17]	Second	Gefitinib (unselected)	0.80	NA
INTEREST Phase III [18]	Second	Docetaxel	0.28	3.9
ZODIAC Phase III [19]	Second	Docetaxel	0.16	4.4
VITAL Phase III [20]	Second	Docetaxel	0.04	28.4

Bayes’ factors showing how likely one resistance model is over the other, and also how important it is to know which lesion belonged to which patient (correlated), over not knowing (independent) across all studies

Finally, we assessed the size of the resistant fraction under gefitinib treatment in IFUM and IPASS, bottom row of Fig. 2: it is approximately 0.3 in IFUM (EGFR mutant selected Caucasian patients) and 0.2 in IPASS (unselected Asian patients). Furthermore, the within-patient variability in the resistant fraction is similar to the between-patient variability across both studies.

**Fig. 3:**
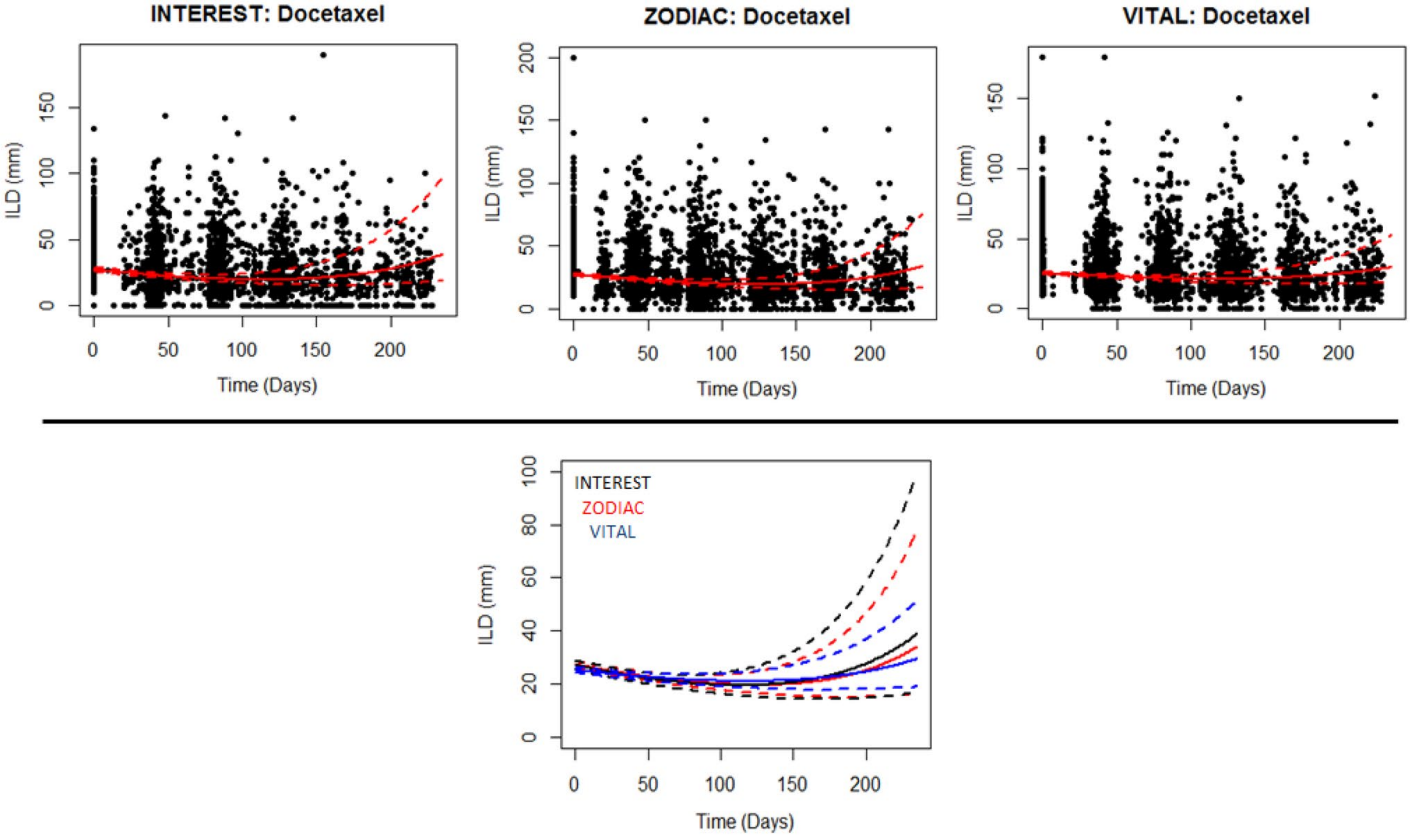
Plots showing the resistance modelling results within the second-line setting. Top row: plots showing the raw individual longest diameter data over time (black dots) together with mean model simulations (solid lines) and 95% confidence intervals (dashed lines) for first-line treatments. Bottom row: comparison of the dynamics via model simulations between the three docetaxel studies in the second-line setting

In the second-line setting for docetaxel, we found the acquired model was generally preferred; most convincingly in VITAL and less convincingly in INTEREST. The mean model simulation from the preferred model together with the 95% CI overlaid on top of the raw data can be seen in the top row of Fig. 3. On comparing the simulations, see bottom row of Fig. 3, we find that the dynamics are consistent and that the uncertainty in the re-growth phase varies between studies; VITAL showing the lowest degree of uncertainty, followed by ZODIAC and then INTEREST.

Three study arms (from IDEAL1, ZEST, and SUNITINIB) with EGFR inhibitors in the second-line setting could also be examined. Although the data could be described well by the model, as shown in Figs. S8–S13, we found that one model was not more likely than the other, in describing the time-series dynamics. Since no winner could be found, further analyses of these treatments were not conducted.

In studies where a preferred model was found, that model was taken forward into the next set of analyses; these explored whether dynamics across lesions, within a patient, were correlated. We found that knowledge of “which lesion belongs to which patient” is important. Thus, there is a degree of correlation in time-series dynamics across lesions, within a patient.

### Autoregressive analysis

**Fig. 4:**
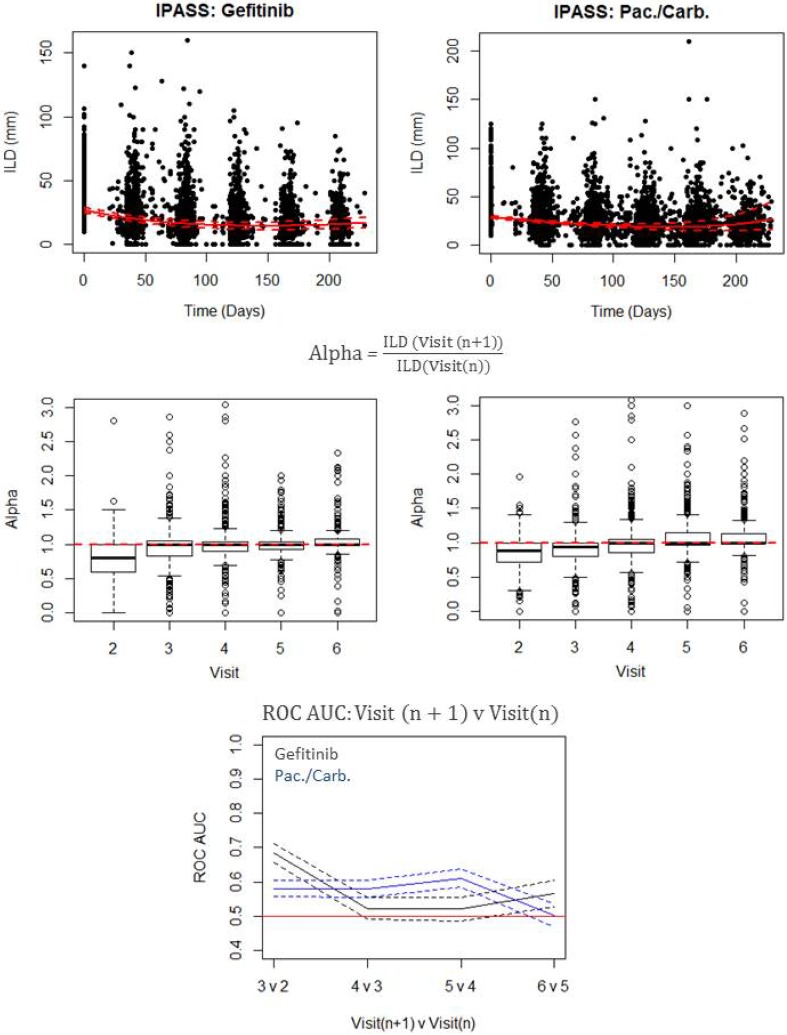
Plots showing the results from the autoregressive analysis. Plots showing the transition from ILD time-series to generation of ROC AUC values over visits for gefitinib and paclitaxel/carboplatin (IPASS study). Top row: ILD time-series with mean model simulations (solid red lines) and 95% CI (red dashed lines) overlaid. Middle row: distributions of alpha values—relative changes between two consecutive visits—moving from one visit to the next for the corresponding raw ILD values shown in the top row. Bottom row: ROC AUC values when using alpha values to discriminate between consecutive visits

In addition to the model-based analyses described to this point, we also assessed whether these differences in dynamics could have been detected visually. The study chosen to assess this was IPASS, since (i) we found that the dynamics were different for the two treatment arms of that study, and (ii) the data collection was similar across the two arms (Supplemental Fig. S15). The process undertaken to analysing the time-series using an autoregressive technique is shown pictorially in Fig. 4.

The raw data with mean model simulations overlaid can be seen in the top row of Fig. 4. The distribution of alpha values, defined as relative change in tumour size from one visit to the next, across visits can be seen in the middle row of Fig. 4. The overall trend in the distribution of alpha values from visit to visit is subtly different. This difference becomes more apparent in the plot at the bottom row of Fig. 4. It shows how well alpha values can discriminate between two consecutive on-treatment visits. For example, at the *3 v 2* point, the ROC AUC values correspond to how well alpha values can discriminate between whether the values are from Visit 2 versus Visit 3. Overall, the dynamics under paclitaxel/carboplatin treatment change continuously until we reach Visit 6. In contrast, for gefitinib, the largest changes in dynamics occur early on; there is then a period of no-change, followed by a subtle change, as we move from Visit 5 to Visit 6. These results show that the dynamics in tumour size, assessed via an autoregressive visualisation approach, are markedly different for gefitinib versus paclitaxel/carboplatin, in further support of the findings using the resistance model competition.

## Discussion

Tumour heterogeneity is known to exist at numerous levels, from between patients to between lesions within a patient, and even within an individual lesion [21–24]. This heterogeneity has been discovered mainly through analyses of tumour material at the microscopic level. The impact of this heterogeneity on the within- and between-patient variability in tumour dynamics, under treatment, in a quantitative way has been largely unexplored. Here, we attempted to analyse these sources of variability by developing a joint mathematical biology and applied statistics approach, for application to routinely collected clinical trial imaging data via RECIST. Given that RECIST classifies lesions in a patient as target or non-target, and since only the former are recorded quantitatively over time, we, therefore, only used target lesions for this analysis. We applied our resultant framework to a sub-group of patients who relapsed from ten clinical studies in non-small cell lung cancer. The studies included chemotherapies (paclitaxel/carboplatin combination, docetaxel) and first-generation EGFR inhibitors (gefitinib, erlotinib), across both first- (treatment naive) and second-line settings. The model-based approach was used to explore what insights can be gleamed regarding treatment resistance after the initial response.

The mathematical models developed here were purposely kept simple and based on our basic understanding of two types of resistance mechanisms as described by Hata et al. [4] The first, termed the de-novo model, is based on the idea that drug treatment selects out a pre-existing resistant clone; the other, termed the acquired model, describes the emergence of a resistant clone while under treatment (note that it is the treatment that dictates the resistance type). The resultant models were tested in a competition framework the same as those used in survival prediction competitions [7], to assess which model best-described tumour size dynamics across all ten study arms.

In the treatment naive setting, we found that the de-novo model best-described the dynamics of tumours under gefitinib treatment, in two independent studies with different patient EGFR mutation selection criteria, IPASS (unselected Asian population) and IFUM (EGFR mutation positive Caucasian population). Using the resultant model, we were able to estimate the fraction of the initial tumour exhibiting resistance, and found this to be similar across both studies; mean resistance fraction was, approximately, 0.3 in IFUM and 0.2 in IPASS. The slight difference in these mean resistant fractions may be due to differences in patient populations between the two studies, e.g., Asians (unselected) versus Caucasians (EGFR mutation positive). These values could be used to inform the development of co-culture xenograft studies, to further explore the dynamic interplay between drug sensitive and resistant cells in preclinical drug development [25].

For treatment naïve patients treated with paclitaxel/carboplatin in contrast to gefitinib, we found that the acquired model best-described tumour size dynamics, in two independent studies. On comparing the dynamics between paclitaxel/carboplatin and gefitinib, we found faster shrinkage and slower re-growth for gefitinib versus doublet chemotherapy, although the shrinkage phase appeared to last longer for doublet chemotherapy. This result highlights how it may be possible to have no significant differences in progression-free survival times between two treatments, yet the dynamics of the tumour size time-series may differ between the two treatments.

The slower re-growth for gefitinib versus paclitaxel/carboplatin suggests that de-novo resistance may lead to a less aggressive resistant clone than acquired resistance. One hypothesis as to why the growth rates of the resistance clones differ between the two resistance modes may lie in how the selection pressure is applied. In the case of gefitinib, the drug is given daily, such that the selection pressure remains constant. This is in contrast to paclitaxel/carboplatin, which is given once every 3 weeks: selection pressure is intermittent, as described previously by Chmielecki et al. [25]. An alternative hypothesis is that gefitinib, as a targeted agent, selects for a specific cell population, whereas paclitaxel/carboplatin does not select out for cells in the same manner [4].

This key finding in the treatment naive setting, namely that tumour size dynamics between gefitinib versus paclitaxel/carboplatin differ, was further explored via a data visualisation approach. This simply involved analysing relative changes in dynamics from one visit to the next, via an ROC analysis. Our results confirmed the key model-based inferences: tumour size dynamics between gefitinib versus paclitaxel/carboplatin differ. The analysis also highlighted, similarly to the modelling exercise, that initial tumour shrinkage may well be greater for gefitinib than paclitaxel/carboplatin. Furthermore, it also indicated that most tumour shrinkage for gefitinib had already occurred by the first visit, 8-week time point, but could still continue for paclitaxel/carboplatin, albeit at a slower rate.

Our results also indicate that, although no further shrinkage is gained from continuing on gefitinib, post 8 weeks, the re-growth rate of the resistant clone appears to be slower than that observed for doublet chemotherapy. This result supports the clinical findings that treating with gefitinib post-progression may still provide patients with a benefit [26].

Application of the resistance model competition to second-line treatments, where patients have previously received chemotherapy combinations, also led to contrasting inferences for docetaxel versus gefitinib or erlotinib. For docetaxel, we found that the acquired model best-described tumour size dynamics, although the strength of this inference varied across the three studies analysed.

On further analysis of the docetaxel studies, we found that the strength of evidence for the acquired hypothesis correlated with the chronological order patients were recruited to studies: INTEREST (weakest evidence—the oldest study: patient recruitment was between March 2004 and February 2006), followed by ZODIAC (patient recruitment was between May 2006 and April 2008), then VITAL (strongest evidence—the most recent study: patients recruited between September 2007 and February 2010).

As we move from the time period when INTEREST was conducted through to VITAL, prior treatments had changed. Of the patients recruited to INTEREST, the oldest study, none had previously taken bevacizumab, as part of a combination therapy and the only platinum based combination used was paclitaxel. As we move on to ZODIAC a small proportion of patients had begun to receive bevacizumab with doublet chemotherapy (3%) with gemcitabine also emerging as a combination partner. Finally, as we move to the latest study, VITAL, approximately 12% of patients had a combination that involved bevacizumab with other platinum combinations involving gemcitabine as well as taxane-based treatments. This changing landscape in treatment history may explain the subtle differences regarding the strength of preference for the acquired model across those studies; selection pressure from the previous treatment would have influenced resistance mechanisms.

For second-line gefitinib and erlotinib, we found that neither model was better than the other at describing tumour size dynamics. This result may seem surprising at first, given that the de-novo model was the preferred one for first-line gefitinib, in two independent studies. However, the second-line patients considered here were given chemotherapy combinations as a first-line treatment; for first-line paclitaxel/carboplatin, the acquired model was preferred, which we interpreted as producing a more aggressive resistant phenotype. Thus, one hypothesis for our observation here is that administration of chemotherapy combinations first followed by a targeted EGFR inhibitor makes it harder to select out a specific clone. This result may suggest that patients for whom targeted therapies are an option should receive these first, before resorting to using chemotherapy.

For the final selected models across both first- (treatment naive) and second-line settings, we also assessed if there was a degree of correlation in tumour size dynamics within a patient. We found that knowledge of “which lesion belongs to which patient” makes a considerable difference in each of the selected models’ ability to describe the data. This result suggests that there is a degree of correlation in tumour size dynamics across metastatic lesions, within a patient. Thus, there is a degree of homogeneity within a patient when it comes to response and relapse of their metastatic lesions, in spite of the known genomic heterogeneity across those lesions [27].

The model-based approach taken within this study is not without caveats. The key one being our approach does not provide any molecular basis of resistance mechanisms, although it would be of interest to combine this approach in a quantitative study where circulating tumour DNA is being analysed longitudinally. We also have not considered recorded data on non-target lesions and new lesions. However, the approach does show that more can be gleamed from routinely collected clinical trial imaging data, and that this could be useful to differentiate between drugs and also assist in developing preclinical experiments. It is also noted that because longest diameter is used as a surrogate the modelling reported here is dependent upon that data. It would be useful if, in future trials, more detailed image analysis was performed including measuring tumour volume.

In summary, our approach and results show that exploring clinical trial imaging data in more detail than simply analysing the sum of longest diameters of the target lesions can lead to important biological and treatment insights. Treatment naïve patients will present with heterogeneous lesions and the choice of treatment will dictate the selection pressure and resulting outgrowing fraction. This will have consequences for the following lines of treatment. We are not aware of a comparable model-based study in the literature. We, therefore, encourage the scientific community to explore the tumour imaging data for more than just searching for a drug independent survival model given that our results show that the tumour dynamics are treatment-dependent; thereby, survival is likely as well.

## Electronic supplementary material

Below is the link to the electronic supplementary material.

Supplementary material 1 (DOCX 1407 kb)
