# Supplementary material for: Resistance models to EGFR inhibition and chemotherapy in non-small cell lung cancer via analysis of tumour size dynamics
Source: Cancer Chemother Pharmacol. 2019 Apr 24;84(1):51–60. doi: 10.1007/s00280-019-03840-3 (PMC6561994; doi:10.1007/s00280-019-03840-3)
Supplement: Supplementary file 1 — Supplementary material 1 (DOCX 1407 kb) [file 280_2019_3840_MOESM1_ESM.docx]

**Supplemental Material**

**Methods**

**Derivation of models of resistance**

We considered two types of resistance mechanisms, *de-novo* and *acquired*, which we here describe in more details. We also show how the *de-novo* model framework can be used to derive two analytical models of tumour growth that are routinely used.

***De-novo* resistance**

Here we assume that there are two cell populations that make up the longest diameter growing over time, $Y_{1}(t)$ and$Y_{2}(t)$, prior to treatment. Under treatment, one population, $Y_{1}$, dies out, at a rate $d$, and the other population, $Y_{2}$, continues to grow at the same rate it was growing prior to treatment, $g$. We shall refer to $Y_{1}$ as the drug sensitive cell population and $Y_{2}$ as the drug resistant cell population. The pair of differential equations describing the rate of change of these cell populations is given by,

$$\dot{Y_{1}}=-dY_{1} (1)$$

and

$$\dot{Y_{2}}=gY_{2}. (2)$$

The temporal evolution of the total cell population, $Y(t)$, is thus given by summing up the integral of the two equations above,

$$Y\left( t \right)=Y_{1}\left( 0 \right)e^{-dt}+Y_{2}\left( 0 \right)e^{gt}, (3)$$

where $Y_{1}\left( 0 \right)$ and $Y_{2}\left( 0 \right)$ are the initial values of, respectively, the drug sensitive and drug resistant cell populations. Overall, there are four parameters that need to be estimated.

**Relationship to existing analytical models of tumour growth**

The two most common models of tumour growth that have been used extensively to describe the temporal evolution of tumour size are: (i) exponential decay and growth given by,

$$Y\left( t \right)=e^{-dt}+e^{gt}-1, (4)$$

which is fit to the relative (*Y(t)/Y(0))*change in tumour size over time and not the absolute value; and (ii) exponential decay and linear growth,

$$Y\left( t \right)=Y\left( 0 \right)e^{-dt}+gt, (5)$$

which is fit to the absolute value of tumour size. Both of these models can be derived using the *de-novo* framework as we now discuss.

For the exponential decay and growth model, equation (4) becomes - assuming, in (3), that $Y_{1}\left( 0 \right)= Y_{2}\left( 0 \right)$,

$$Y\left( t \right)=Y_{1}\left( 0 \right){(e}^{-dt}+e^{gt}), (6)$$

with $Y\left( 0 \right)= {2Y}_{1}\left( 0 \right)$. Next we divide through by $Y_{1}\left( 0 \right),$

$$\frac{Y(t)}{Y_{1}\left( 0 \right)}=\frac{1}{2}\left( e^{-dt}+e^{gt} \right). (7)$$

We see that the right-hand side is very similar to equation (4), without the constant *-1*. The introduction of *-1* to the right hand-side of (4) (which ensures Y(t)/Y(1)=1 at t=0) implies that, at *t=0*, either the whole of the tumour is drug resistant or sensitive. The original paper by Stein *et al*. would suggest the latter, *i.e.*, the whole of the tumour is in the drug sensitive state. Therefore, the *-1* simply reduces the resistant fraction to *0* at *t=0* and, thus, the model implies the resistance appears instantly as soon as treatment is given. The key assumption to derived equation (4) from (3) is therefore that the entire tumour is drug sensitive, but a resistant clone develops instantly, as the drug is given.

The derivation of the exponential decay and linear growth from equation (4) can be achieved by assuming that tumour growth is not a first order process, but zero order, *i.e*., the process of tumour shrinkage is different to that of growth. Therefore, equation (2) becomes,

$$\dot{Y_{2}}=g, (8)$$

and (3) becomes,

$$Y\left( t \right)=Y_{1}\left( 0 \right)e^{-dt}+Y_{2}\left( 0 \right)+gt. (9)$$

Next, if we assume that (i) there are no cells, initially, resistant to drug treatment, $Y_{2}\left( 0 \right)=0$, and (ii) all cells are sensitive to the drug, then (9) becomes,

$$Y\left( t \right)=Y\left( 0 \right)e^{-dt}+gt, (10)$$

which is the same as equation (5). The key assumptions were:

1. process of tumour shrinkage is different to tumour growth, i.e., shrinkage is first order and growth is zero order,
2. no cells are resistant to the drug prior to treatment, but we have spontaneous production of resistant cells at treatment onset,
3. all cells are initially sensitive to the drug.

Having established a model for *de-novo* resistance and ascertained that the current literature models are special cases of the general *de-novo* model, we now develop a model for acquired resistance.

***Acquired* resistance**

For the *acquired* resistance model, we assume that all cells are affected by the drug, albeit in two different ways: they either die, or adapt to become resistant and subsequently proliferate. We define $Y_{1}(t)$ as the cell population that can either die under treatment, at a rate $d$, or adapt, at a rate $c$, and join cell population $Y_{2}(t)$which grows, at a rate $g$. The rates of change of $Y_{1}(t)$ and $Y_{2}\left( t \right)$ are given by the following pair of differential equations,

$$\dot{Y_{1}}=-(d+c)Y_{1} (11)$$

and

$$\dot{Y_{2}}=cY_{1}+gY_{2}. (12)$$

We assume that, prior to treatment, there are no cells in state $Y_{2}$. The temporal evolution of the total cell population, $Y(t)$, is thus given by summing up the integral of the two equations above,

$$Y\left( t \right)=Y_{1}\left( 0 \right)e^{-(d+c)t}+\frac{cY_{1}(0)}{d+c+g}{(e}^{gt}-e^{-(d+c)t}), (13)$$

where $Y_{1}\left( 0 \right)$ is the initial value of the combined drug sensitive and adaptive cell population. Similarly to the *de-novo* model, there are only four parameters that are required to be estimated.

Re-arranging the *acquired* model, equation (13), gives,

$$Y\left( t \right)=Y_{1}\left( 0 \right)\left( 1-\frac{c}{d+c+g} \right)e^{-\left( d+c \right)t}+\frac{cY_{1}\left( 0 \right)}{d+c+g}e^{gt}, (14)$$

**Comparison of models**

When the de-novo and the acquired resistance models are compared (equations 3 and 14) we see that qualitatively the models give the same dynamics; they are both sum of exponentials but the parameterisations are different. This difference in parameterisation can lead to different models being preferred depending on the kinetics of the data which we shall now demonstrate via a simulation study.

Two sets of *de-novo* model population parameters were chosen to give differing dynamics for the simulation study see Table S1.

**Table S1:** simulation study parameter sets.

|  | log(Y1(0)) – (%CV) | log(Y2(0)) – (%CV) | log(g) – (%CV) | log(d) – (%CV) | R.E. - S.D. |
| --- | --- | --- | --- | --- | --- |
| Set 1 | 3.50 (20) | -3 (20) | -4.5 (20) | -5 (20) | 1 |
| Set 2 | 3.25 (20) | 2 (20) | -5.5 (20) | -4 (20) | 1 |

%CV – percent coefficient of variation; R.E. - (Additive) Residual Error; S.D. – Standard Deviation

When comparing parameter sets 1 and 2 in Table S1 we can see that,

1. Set 1 assumes that the lesion contains a small but fast growing (resistant) fraction compared to Set 2.
2. With respect to the decaying fraction we see that this is larger and decays faster in parameter Set 2 than Set 1.

We simulated 500 lesions using the parameter distributions in Table S1 in the following way.

For each lesion we drew a set of parameters from the distribution described in Table S1. With this set of parameters we used the *de-novo* model to simulate a time-course of lesion size measurements at 56 day intervals until a lesion had grown by 20% over the minimum. Note at each time-point noise was added to the measurement by adding a value drawn from the normal distribution with mean zero and variance one (additive residual error). (It must be noted that this simulation protocol is not equivalent to how the data is collected via RECIST. Imaging time-series drop-outs can be based on one of 7 possible progression categories as described in the main article.)

We then fitted both the acquired and de-novo model to each data-set created assuming additive residual error, the BIC values for each model for each data-set can be seen in Table S2. The table clearly shows that for Set 1 the *acquired* model is favoured over the *de-novo* model and for Set 2 the inference is the opposite i.e. the *de-novo* model is favoured over the *acquired*.

**Table S2:** simulation study BIC values using *acquired* and *de-novo* models.

|  | *Acquired* BIC | *De-novo* BIC |
| --- | --- | --- |
| Set 1 | 24532 | 43654 |
| Set 2 | 20014 | 15958 |

Table S3 shows the parameter values when fitting each model to each of the simulated data-sets.

For Set 1 we found the variance components for *d* and *Y_2_(0)* shrunk to zero for the *de-novo* model. Note, that the estimate of the fixed effect for *Y_2_(0)* is also highly uncertain, confidence intervals include zero, and quite far from the original value of -3. In comparison the parameter estimates for the *acquired* model, for Set 1, are well estimated. Furthermore if we calculate the growing fraction using,

$$\frac{cY_{1}(0)}{d+c+g} (15)$$

we find the logarithm of this value to be, -3.22; close to the original value, -3, used in the simulation.

For Set 2 we found the opposite results to those for Set 1. Namely that the variance components of two of the parameters, *d* and *c*, shrunk to zero for the *acquired* model. Furthermore we found that the estimates of, *g* and *d*, were further away from the original values than those estimated using the *de-novo* model.

The brief simulation study results highlight how differences within the kinetics in the time-series can lead to different models being preferred.

**Table S3:** parameter values for *acquired* and *de-novo* models from the simulated data-sets with 95% confidence intervals (95% C.I.).

| *De-novo* | | *log(Y_1_(0))* | *log(d)* | *log(Y_2_(0))* | *log(g)* |
| --- | --- | --- | --- | --- | --- |
| Set 1 | Fixed Effects  (95% C.I.) | 3.61  (3.54, 3.67) | -5.32  (-5.42, -5.21) | -0.90  (-2.13, 0.33) | -6.03  (-6.59, -5.47) |
|  | S.D.  (95% C.I.) | 0.59  (0.55, 0.64) | 0 | 0 | 0.78  (0.70, 0.87) |
| Set 2 | Fixed Effects  (95% C.I.) | 3.28  (3.23, 3.34) | -4.15  (-4.23, -4.07) | 1.81  (1.74, 1.88) | -5.17  (-5.27, -5.08) |
|  | S.D.  (95% C.I.) | 0.58  (0.54, 0.62) | 0.77  (0.72, 0.83) | 0.49  (0.43, 0.54) | 0.77  (0.72, 0.84) |
|  | | | | | |
| *Acquired* | | *log(Y_1_(0))* | *log(d)* | *log(c)* | *log(g)* |
| Set 1 | Fixed Effects  (95% C.I.) | 3.47  (3.41, 3.53) | -4.39  (-4.47, -4.30) | -10.63  (-10.81, -10.44) | -4.95  (-5.04, -4.87) |
|  | S.D.  (95% C.I.) | 0.71  (0.66, 0.75) | 0.92  (0.86, 0.99) | 1.36  (1.18, 1.56) | 0.95  (0.89, 1.01) |
| Set 2 | Fixed Effects  (95% C.I.) | 3.55  (3.51, 3.59) | -4.53  (-4.59, -4.48) | -5.59  (-5.71, -5.48) | -5.32  (-5.49, -5.14) |
|  | S.D.  (95% C.I.) | 0.45  (0.42, 0.48) | 0 | 0 | 1.54  (1.38, 1.72) |

S.D. - standard deviation.

**Example Code**

We first need to set-up the groupedData object in *nlme*. For one level of hierarchy, assuming each lesion has its own identifier, the following code is used to set-up the groupedData object,

dat.grp<-groupedData(LD~TIME|LESION,data=dat)

where dat is a data frame which has columns, PATIENT, LESION, LD and TIME. PATIENT is the patient identifier, LESION is the lesion identifier, LD is the longest diameter and TIME is the time-point at which the lesion measurement was taken. For two levels of hierarchy i.e. accounting for which lesion belongs to which patient the following groupedData object is required,

dat.grp<-groupedData(LD~TIME|PATIENT/LESION,data=dat)

Having established the groupedData object we can then fit the “acquired” model to the data using the following piece of code

fit1<-nlme(LD~exp(a)*exp(-(exp(b)+exp(c))*TIME)+

(exp(c)*exp(a)/(-exp(b)-exp(c)-exp(d)))*

(-exp(exp(d)*TIME)+exp((-exp(b)-exp(c))*TIME)),

fixed = a+b+c+d~1,

random = pdDiag(a+b+c+d~1),

data = dat.grp,

start = c(4,-4,-7,-4),

method='ML',

verbose = TRUE,

control = nlmeControl(pnlsMaxIter=10,tolerance=1e-3))

The expression to the right of ~ is the formula for the “acquired” model, see derivation earlier in this document. After the declaration of the model, we declare the fixed and random effects, followed by the data , initial starting values for the fixed effects, method, whether we want to see the output of each iteration and finally the tolerances. The code required to fir the “de-novo” model is as follows,

fit2<-nlme(LD~exp(a)*exp(exp(b)*TIME)+exp(c)*exp(-exp(d)*TIME),

fixed = a+b+c+d~1,

random = pdDiag(a+b+c+d~1),

data = dat.grp,

start = c(2,-5,3,-4),

method='ML',

verbose = TRUE, control = nlmeControl(pnlsMaxIter=10,tolerance=1e-3))

**Results**

The diagnostic plots correspond to the final model for gefitinib and carboplatin / paclitaxel, when accounting for both between- and within-patient variabilities in tumour lesion dynamics. Parameter estimates are detailed in the tables along with 95% confidence intervals (C.I.). C.I. are not reported for parameters that are fixed, for example random effects with standard deviation 0.

**Diagnostic plots and parameter values**

**1^st^ Line**

**Paclitaxel/Carboplatin (IPASS) – Acquired Model**


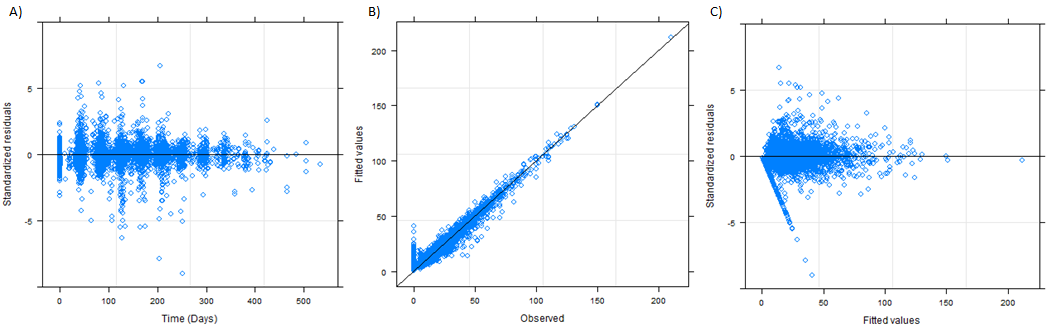


**Figure S1:** Diagnostic plots for the final model: A) time *vs*. individual standardised residuals; B) observed *vs*. individual fitted; C) individual fitted *vs*. standardised residuals

**Table S4:** Parameter estimates for the final model, with 95% bootstrapped confidence intervals (*C.I.*)

|  | *log(Y_1_(0))* | *log(d)* | *log(c)* | *log(g)* |
| --- | --- | --- | --- | --- |
| Fixed Effects  (95% C.I.) | 3.35  (3.31, 3.38) | -5.47  (-5.56, -5.38) | -8.62  (-9.00, -8.23) | -4.00  (-4.11, -3.88) |
| S.D. Patient Level  (95% C.I.) | 0.17  (0.12, 0.23) | 0.64  (0.57, 0.73) | 0.98  (0.83, 1.15) | 0.64  (0.57, 0.73) |
| S.D. Lesion Level  (95% C.I.) | 0.47  (0.44, 0.49) | 0.42  (0.37, 0.48) | 0  NA | 0.12  (0.10, 0.14) |

**Paclitaxel/Carboplatin (ABRAXANE) – Acquired Model**


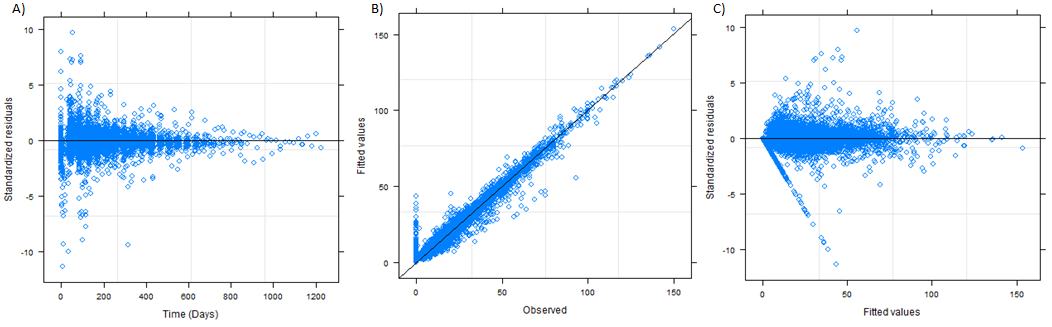


**Figure S2:** Diagnostic plots for the final model: A) time *vs*. individual standardised residuals; B) observed *vs*. individual fitted; C) individual fitted *vs*. standardised residuals

**Table S5:** Parameter estimates for the final model, with 95% bootstrapped confidence intervals (*C.I.*)

|  | *log(Y_1_(0))* | *log(d)* | *log(c)* | *log(g)* |
| --- | --- | --- | --- | --- |
| Fixed Effects  (95% C.I.) | 3.28  (3.23, 3.31) | -5.08  (-5.31, -4.99) | -6.79  (-8.17, -6.09) | -4.89  (-5.26, -4.29) |
| S.D. Patient Level  (95% C.I.) | 0.24  (0.21, 0.29) | 1.04  (0.54, 1.14) | 1.92  (0.75, 3.22) | 1.05  (0, 1.39) |
| S.D. Lesion Level  (95% C.I.) | 0.51  (0.49, 0.55) | 0.62  (0.50, 0.73) | 0.72  (0,1.00) | 0  NA |

**Gefitinib (IPASS) – *De-novo* Model**


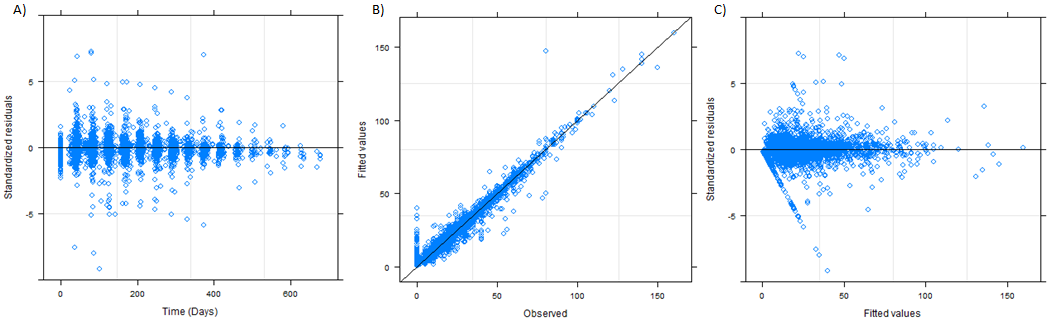


**Figure S6:** Diagnostic plots for the final model: A) time *vs*. individual standardised residuals; B) observed *vs*. individual fitted; C) individual fitted *vs*. standardised residuals

**Table S7:** Parameter estimates for the final model, with 95% bootstrapped confidence intervals (*C.I.*)

|  | *log(Y_1_(0))* | *log(d)* | *log(Y_2_(0))* | *log(g)* |
| --- | --- | --- | --- | --- |
| Fixed Effects  (95% C.I.) | 3.06  (2.99, 3.12) | -4.47  (-4.58, -4.35) | 1.67  (1.53, 1.81) | -5.42  (-5.56, -5.28) |
| S.D. Patient Level  (95% C.I.) | 0.24  (0, 0.30) | 0.64  (0.55, 0.78) | 0.63  (0.53, 1.12) | 0.90  (0.78, 1.00) |
| S.D. Lesion Level  (95% C.I.) | 0.50  (0.44, 0.55) | 0.49  (0.35, 0.63) | 0.68  (0.64, 1.16) | 0.11  (0, 0.41) |

**Gefitinib (IFUM) – *De-novo* Model**


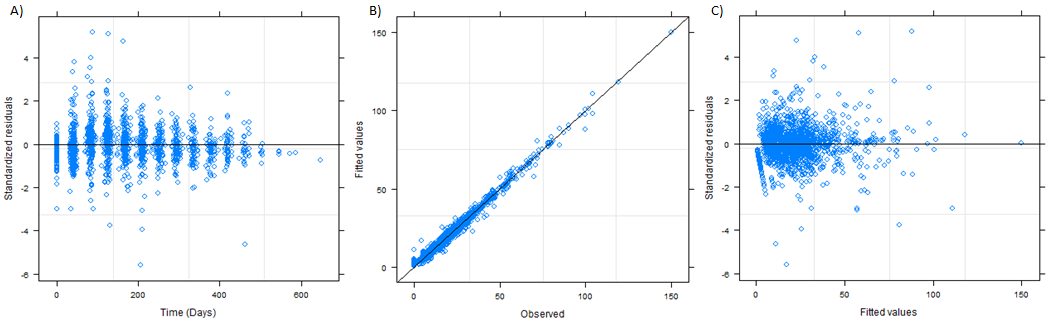


**Figure S4:** Diagnostic plots for the final model: A) time *vs*. individual standardised residuals; B) observed *vs*. individual fitted; C) individual fitted *vs*. standardised residuals

**Table S8:** Parameter estimates for the final model, with 95% bootstrapped confidence intervals (*C.I.*)

|  | *log(Y_1_(0))* | *log(d)* | *log(Y_2_(0))* | *log(g)* |
| --- | --- | --- | --- | --- |
| Fixed Effects  (95% C.I.) | 2.90  (2.80, 3.01) | -4.10  (-4.27, -3.93) | 1.91  (1.65, 2.17) | -6.39  (-6.71, -6.07) |
| S.D. Patient Level  (95% C.I.) | 0.31  (0.20, 0.49) | 0.57  (0.42, 0.78) | 0.97  (0.76, 1.24) | 1.18  (0.93, 1.49) |
| S.D. Lesion Level  (95% C.I.) | 0.54  (0.47, 0.62) | 0.58  (0.48, 0.72) | 0.87  (0.74, 1.01) | 0.30  (0.18, 0.50) |

**2^nd^ Line**

**Winner**

**Docetaxel (INTEREST) – Acquired Model**


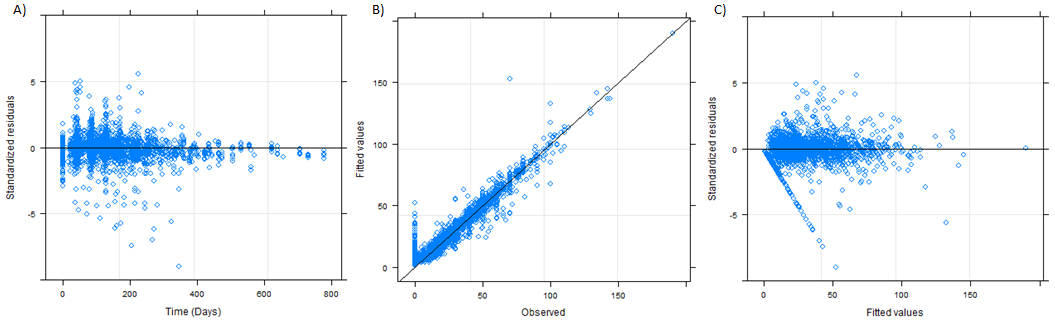


**Figure S5** Shows the diagnostic plots for the final model: A) time *vs*. individual standardised residuals; B) observed *vs*. individual fitted; C) individual fitted *vs*. standardised residuals

**Table S8** Showing parameter estimates for the final model, with 95% bootstrapped confidence intervals (*C.I.*)

|  | *log(Y_1_(0))* | *log(d)* | *log(c)* | *log(g)* |
| --- | --- | --- | --- | --- |
| Fixed Effects  (95% C.I.) | 3.32  (3.27, 3.37) | -5.50  (-5.65, -5.25) | -7.78  (-8.37, -7.20) | -4.07  (-4.27, -3.88) |
| S.D. Patient Level  (95% C.I.) | 0.27  (0.21, 0.33) | 0.72  (0.59, 0.89) | 2.26  (1.81, 2.83) | 0.56  (0.44, 0.71) |
| S.D. Lesion Level  (95% C.I.) | 0.51  (0.48, 0.55) | 0.72  (0.63, 0.82) | 0.23  (0.05, 1.11) | 0.12  (0.06, 0.24) |

**Docetaxel (ZODIAC) – Acquired Model**


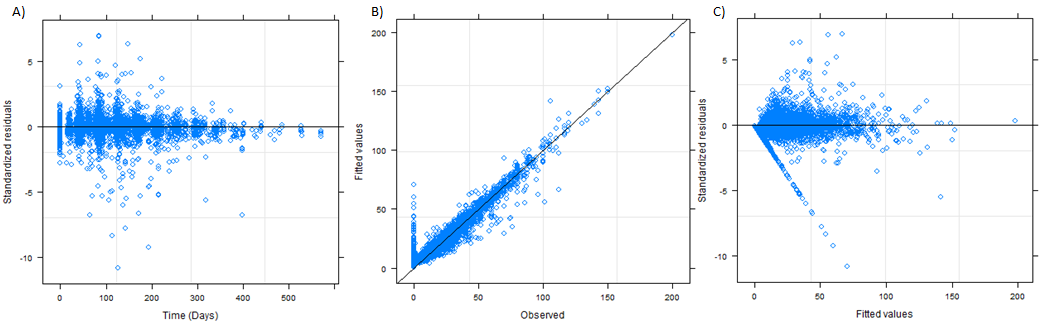


**Figure S6** Shows the diagnostic plots for the final model: A) time *vs*. individual standardised residuals; B) observed *vs*. individual fitted; C) individual fitted *vs*. standardised residuals

**Table S9** Showing parameter estimates for the final model, with 95% bootstrapped confidence intervals (*C.I.*)

|  | *log(Y_1_(0))* | *log(d)* | *log(c)* | *log(g)* |
| --- | --- | --- | --- | --- |
| Fixed Effects  (95% C.I.) | 3.31  (3.27, 3.36) | -5.51  (-6.18, -5.09) | -7.71  (-8.24, -7.18) | -4.19  (-5.39, -0.07) |
| S.D. Patient Level  (95% C.I.) | 0.20  (0.15, 0.28) | 0.70  (0.58, 0.84) | 0.82  (0.50, 1.35) | 0.52  (0.45, 0.60) |
| S.D. Lesion Level  (95% C.I.) | 0.51  (0.48, 0.55) | 0.38  (0.31, 0.47) | 0  NA | 0.11  (0.01, 0.15) |

**Docetaxel (VITAL) – Acquired Model**


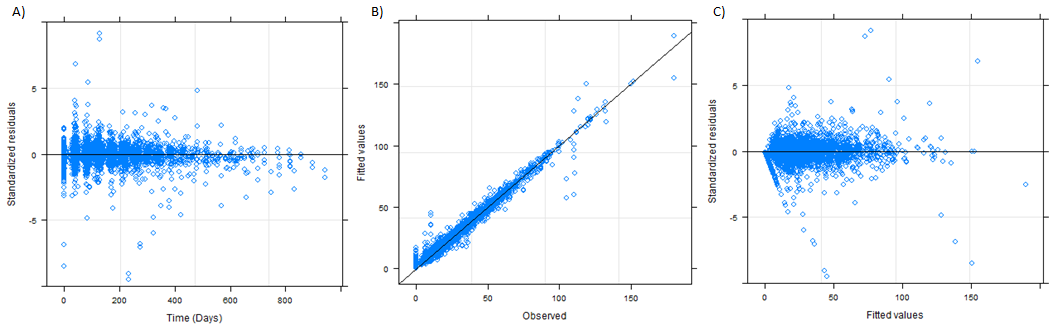


**Figure S7** Shows the diagnostic plots for the final model: A) time *vs*. individual standardised residuals; B) observed *vs*. individual fitted; C) individual fitted *vs*. standardised residuals

**Table S10** Showing parameter estimates for the final model, with 95% bootstrapped confidence intervals (*C.I.*)

|  | *log(Y_1_(0))* | *log(d)* | *log(c)* | *log(g)* |
| --- | --- | --- | --- | --- |
| Fixed Effects  (95% C.I.) | 3.23  (3.19, 3.27) | -5.98  (-6.14, -5.83) | -7.30  (-7.76, -6.85) | -4.48  (-4.69, -4.28) |
| S.D. Patient Level  (95% C.I.) | 0.20  (0.15, 0.27) | 0.84  (0.71, 0.99) | 1.46  (1.18, 1.80) | 0.84  (0.73, 0.97) |
| S.D. Lesion Level  (95% C.I.) | 0.51  (0.48, 0.54) | 0.67  (0.59, 0.77) | 0.41  (0.17, 1.00) | 0.12  (0.02, 0.71) |

**No Winner**

**Gefitinib (IDEAL1) – Acquired Model**


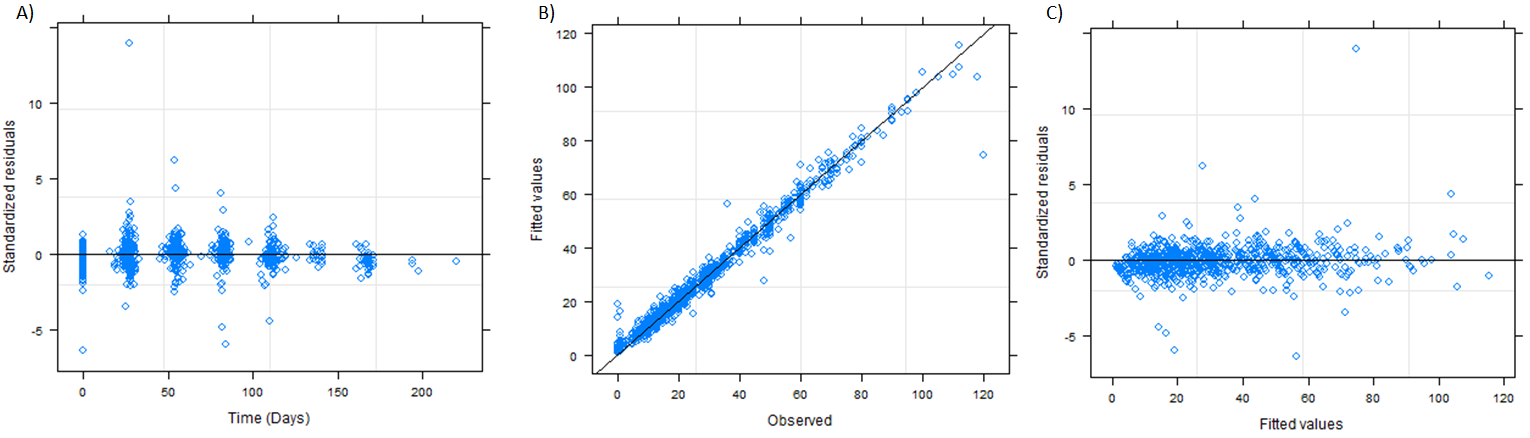


**Figure S8** Shows the diagnostic plots for the final model: A) time *vs*. individual standardised residuals; B) observed *vs*. individual fitted; C) individual fitted *vs*. standardised residuals

**Table S11** Showing parameter estimates for the final model, with 95% bootstrapped confidence intervals (*C.I.*)

|  | *log(Y_1_(0))* | *log(d)* | *log(c)* | *log(g)* |
| --- | --- | --- | --- | --- |
| Fixed Effects  (95% C.I.) | 3.41  (3.31, 3.51) | -5.00  (-5.29, -4.72) | -5.36  (-5.97, -4.74) | -4.41  (-4.77, -4.05) |
| S.D. Patient Level  (95% C.I.) | 0.35  (0.26, 0.47) | 1.07  (0.80, 1.42) | 1.48  (0.89, 2.45) | 0.83  (0.55, 1.26) |
| S.D. Lesion Level  (95% C.I.) | 0.52  (0.46, 0.59) | 0.68  (0.53, 0.88) | 1.14  (0.59, 2.23) | 0  NA |

**Gefitinib (IDEAL1) – *De-novo* Model**


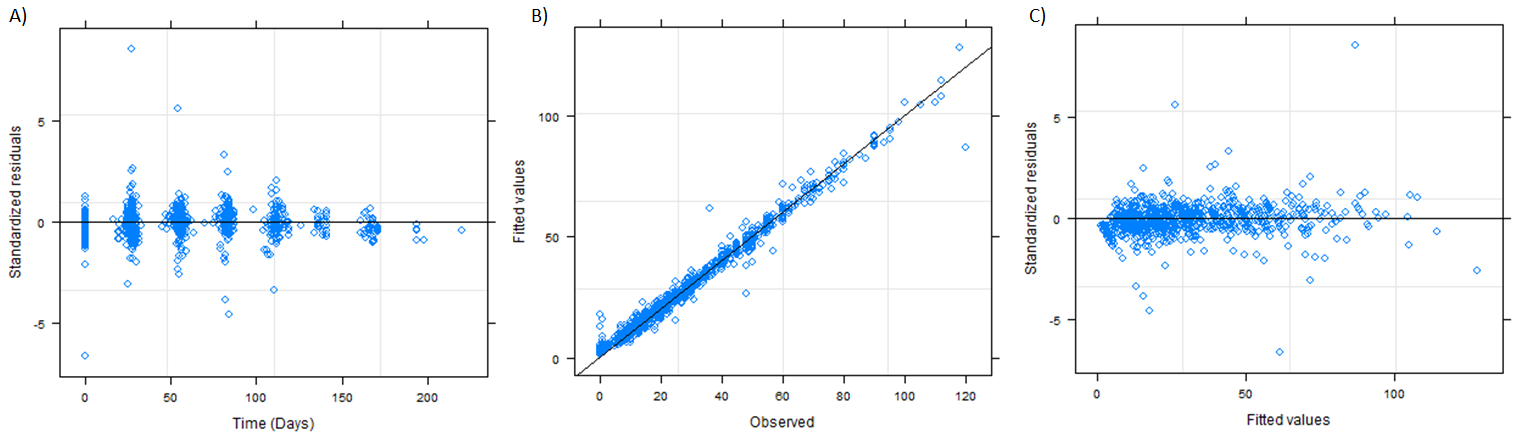


**Figure S9** Shows the diagnostic plots for the final model: A) time *vs*. individual standardised residuals; B) observed *vs*. individual fitted; C) individual fitted *vs*. standardised residuals

**Table S12** Showing parameter estimates for the final model, with 95% bootstrapped confidence intervals (*C.I.*)

|  | *log(Y_1_(0))* | *log(d)* | *log(Y_2_(0))* | *log(g)* |
| --- | --- | --- | --- | --- |
| Fixed Effects  (95% C.I.) | 3.21  (3.09, 3.33) | -4.63  (-4.88, -4.37) | 1.39  (0.97, 1.80) | -4.26  (-4.46, -4.05) |
| S.D. Patient Level  (95% C.I.) | 0.31  (0.21, 0.47) | 0.74  (0.52, 1.05) | 0.85  (0.64, 1.12) | 0.4  (0.29, 0.56) |
| S.D. Lesion Level  (95% C.I.) | 0.54  (0.47, 0.61) | 0.63  (0.48, 0.82) | 0.57  (0.46, 0.69) | 0  NA |

**Erlotinib (ZEST) – Acquired Model**


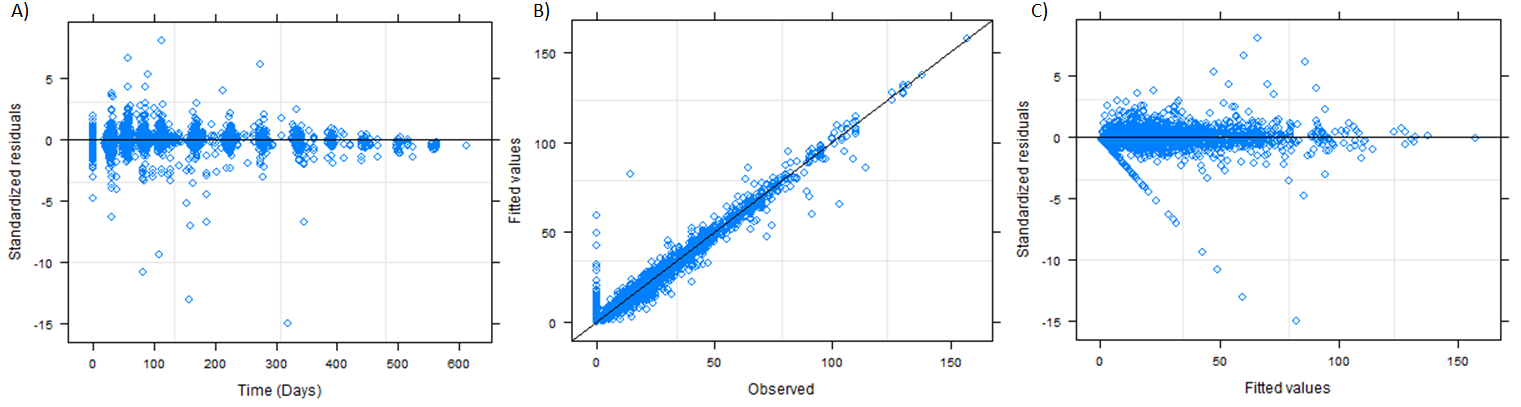


**Figure S10** Shows the diagnostic plots for the final model: A) time *vs*. individual standardised residuals; B) observed *vs*. individual fitted; C) individual fitted *vs*. standardised residuals

**Table S13** Showing parameter estimates for the final model, with 95% bootstrapped confidence intervals (*C.I.*)

|  | *log(Y_1_(0))* | *log(d)* | *log(c)* | *log(g)* |
| --- | --- | --- | --- | --- |
| Fixed Effects  (95% C.I.) | 3.26  (3.21, 3.32) | -5.37  (-5.57, -5.16) | -6.32  (-6.72, -5.92) | -5.24  (-5.50, -4.98) |
| S.D. Patient Level  (95% C.I.) | 0.18  (0.08, 0.34) | 1.08  (0.74, 1.84) | 1.20  (0, 9.4) | 0.80  (0.68, 1.88) |
| S.D. Lesion Level  (95% C.I.) | 0.53  (0.48, 0.65) | 0.71  (0.53, 0.96) | 0  NA | 0.28  (0, 0.43) |

**Erlotinib (ZEST) – *De-novo* Model**


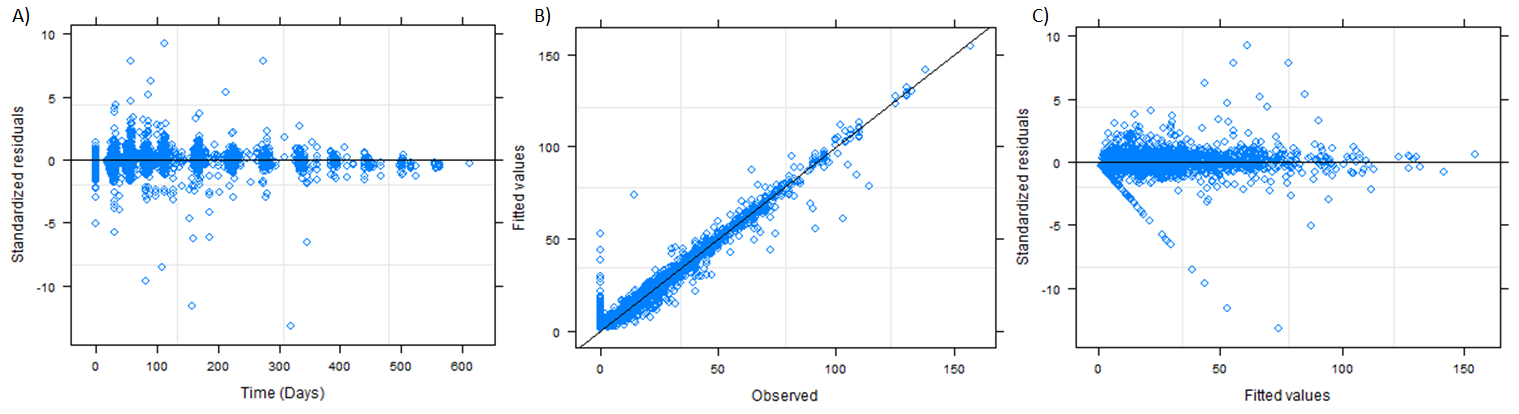


**Figure S11** Shows the diagnostic plots for the final model: A) time *vs*. individual standardised residuals; B) observed *vs*. individual fitted; C) individual fitted *vs*. standardised residuals

**Table S14** Showing parameter estimates for the final model, with 95% bootstrapped confidence intervals (*C.I.*)

|  | *log(Y_1_(0))* | *log(d)* | *log(Y_2_(0))* | *log(g)* |
| --- | --- | --- | --- | --- |
| Fixed Effects  (95% C.I.) | 2.76  (2.67, 2.85) | -4.45  (-4.64, -4.27) | 2.12  (1.96, 2.29) | -5.99  (-6.19, -5.80) |
| S.D. Patient Level  (95% C.I.) | 0.27  (0.17, 0.43) | 0.98  (0.84, 1.14) | 0.74  (0.58, 0.95) | 0.90  (0.74, 1.08) |
| S.D. Lesion Level  (95% C.I.) | 0.52  (0.47, 0.58) | 0.40  (0.30, 0.54) | 0.84  (0.75, 0.94) | 0.20  (0.11, 0.39) |

**Erlotinib (SUNITINIB) – Acquired Model**


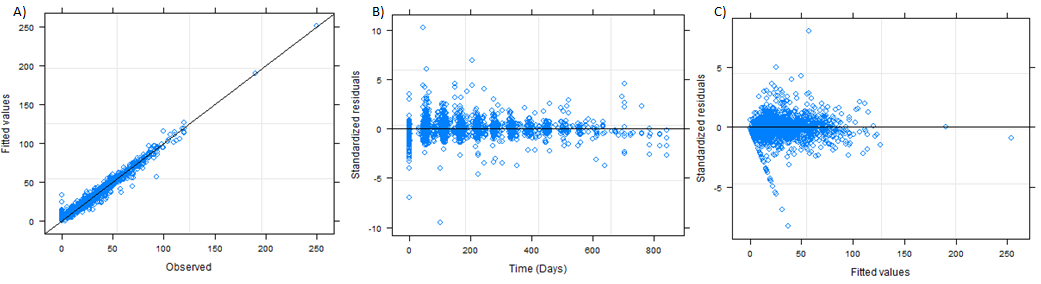


**Figure S12** Shows the diagnostic plots for the final model: A) time *vs*. individual standardised residuals; B) observed *vs*. individual fitted; C) individual fitted *vs*. standardised residuals

**Table S15** Showing parameter estimates for the final model, with 95% bootstrapped confidence intervals (*C.I.*)

|  | *log(Y_1_(0))* | *log(d)* | *log(c)* | *log(g)* |
| --- | --- | --- | --- | --- |
| Fixed Effects  (95% C.I.) | 3.28  (3.22, 3.33) | -6.00  (-6.25, -5.76) | -7.00  (-7.49, -6.51) | -4.58  (-4.82, -4.34) |
| S.D. Patient Level  (95% C.I.) | 0.14  (0.07, 0.26) | 1.19  (0.98, 1.44) | 1.53  (1.21, 1.93) | 0.91  (0.77, 1.08) |
| S.D. Lesion Level  (95% C.I.) | 0.55  (0.51, 0.59) | 0.96  (0.83, 1.10) | 0  NA | 0.33  (0.26, 0.40) |

**Erlotinib (SUNITINIB) – *De-novo* Model**


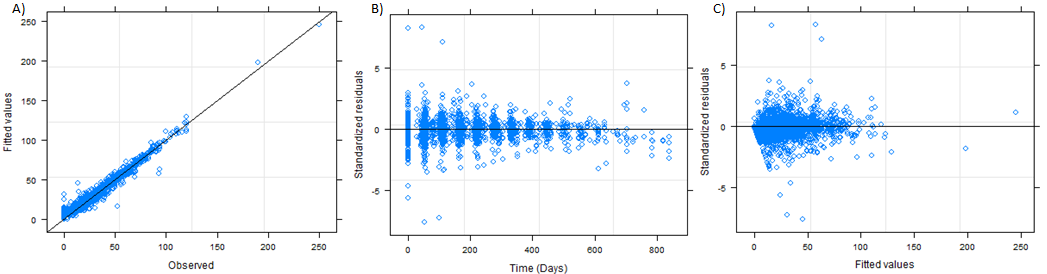


**Figure S13** Shows the diagnostic plots for the final model: A) time *vs*. individual standardised residuals; B) observed *vs*. individual fitted; C) individual fitted *vs*. standardised residuals

**Table S16** Showing parameter estimates for the final model, with 95% bootstrapped confidence intervals (*C.I.*)

|  | *log(Y_1_(0))* | *log(d)* | *log(Y_2_(0))* | *log(g)* |
| --- | --- | --- | --- | --- |
| Fixed Effects  (95% C.I.) | 2.95  (2.87, 3.03) | -5.51  (-5.74, -5.28) | 1.39  (1.13, 1.64) | -5.11  (-5.19, -5.04) |
| S.D. Patient Level  (95% C.I.) | 0.26  (0.17, 0.40) | 1.22  (0.17, 0.40) | 1.28  (1.07, 1.52) | 0  NA |
| S.D. Lesion Level  (95% C.I.) | 0.63  (0.58, 0.68) | 0  NA | 0.72  (0.63, 0.82) | 0  NA |

**Data Collection**

Figure S14 shows the frequency of data points over time for individual lesions, from the gefitinib (pink) and paclitaxel/carboplatin (blue) arms of IPASS.


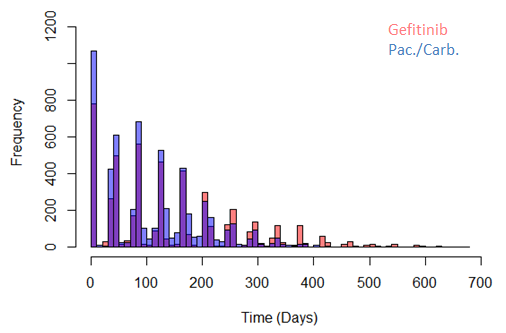


Figure S14: Histogram showing the collection of tumour size measurements over time, for gefitinib and paclitaxel/carboplatin from the IPASS study.
